# Draft Genome Sequences of Six Bacteria Isolated from the Benham Bank, Philippine Rise, Philippines

**DOI:** 10.1128/MRA.00777-19

**Published:** 2019-07-18

**Authors:** Saul M. Rojas, Albert Remus R. Rosana, Andrew D. Montecillo, Mark Dondi M. Arboleda, Hildie Maria E. Nacorda, Nacita B. Lantican

**Affiliations:** a Microbiology Division, Institute of Biological Sciences, University of the Philippines Los Baños, Los Baños, Laguna, Philippines; b Department of Chemistry, University of Alberta, Edmonton, Alberta, Canada; c School of Environmental Science and Management, University of the Philippines Los Baños, Los Baños, Laguna, Philippines; University of Southern California

## Abstract

We report here the draft genome sequences of six bacteria isolated from the near-bottom waters and surface sediments of the Benham Bank, Philippine Rise, Philippines. These genome sequences represent candidate novel species and/or strains from the families Flavobacteriaceae and Dermacoccaceae and the genera Idiomarina, Bacillus, and Vibrio.

## ANNOUNCEMENT

The Benham Bank is the shallowest portion (∼50 m deep) of the Philippine Rise, situated at 15°47′36.10″N and 124°17′47.65″E (1). It is covered in clear waters, allowing sunlight to reach its depth, and was previously shown to host a diverse community of hard corals with associated reef fishes, sponges, and algae (1). Except for fishing within the narrow window of March to June every year, this unique offshore coral reef environment is naturally protected from further anthropogenic activities. We hypothesized that the Benham Bank may harbor bacteria that could produce novel bioactive compounds that can be used as antimicrobial or anticancer drugs, fungicides, herbicides, or pesticides. Here, we report the draft genome sequences of six bacteria isolated from the near-bottom waters and surface sediments (∼50-m depth) of Benham Bank. The six bacterial isolates were preserved at the Microbiology Division, Institute of Biological Sciences, University of the Philippines Los Baños, under the accession codes 10Pi, 144Ye, 147Ba, 29L, Br233, and Br345.

Samples from the Benham Bank were obtained by professional divers. Near-bottom water samples were collected and surface sediments were directly scooped from the bottom (∼50-m depth) using sterile conical tubes. The fresh samples were inoculated into marine broth 2216 (Difco Laboratories) and were incubated onboard at 28°C for 48 hours. The inoculum was serially diluted, plated on marine agar 2216, and incubated aerobically at 28°C for 72 hours. Bacterial colonies with distinct morphological characteristics were picked and extensively purified by streak plating, generating single-colony isolates. Genomic DNA was extracted and purified from the bacterial isolates grown overnight in marine broth 2216 (28°C, 180 rpm) using a Wizard genomic DNA purification kit (Promega, USA) following the manufacturer’s protocol. Sequencing libraries (1 ng) were prepared using a Nextera XT DNA library preparation kit (Illumina, San Diego, CA), and sequencing was performed using a MiSeq reagent kit v2 (2 × 250-bp cycles). FASTQC v0.11.8 (http://www.bioinformatics.babraham.ac.uk/projects/fastqc/) was used to inspect the quality of the sequences, and quality trimming based on the FASTQC report was carried out using the Trimmomatic tool (Galaxy v0.36.5) (2) implemented online in Galaxy (https://usegalaxy.org/) (3). Trimmed reads were assembled *de novo* using Unicycler with default parameters (Galaxy v0.4.6.0) (4), and assembly quality was assessed using QUAST v4.6 (5). The draft genome sequences were annotated using the Rapid Annotation using Subsystems Technology (RAST) v2.0 RASTtk pipeline (6, 7), and taxonomic classification of the isolates was established using the Microbial Genomes Atlas (MiGA) v0.3.6.2 workflow (8) analysis by calculating the average nucleotide identity (ANI) and average amino acid identity (AAI) in comparison to those in the NCBI RefSeq and Prokaryote databases. Gene inventories for secondary metabolite production were predicted using antiSMASH v5 (9). All programs were run with default parameters unless otherwise noted. Genome sequence assembly attributes are summarized in Table 1.

**TABLE 1:** Genomic features and accession numbers of the six bacteria isolated from the Benham Bank, Philippine Rise, Philippines

Isolate	GenBank accession no.	SRA accession no.	Closest relative (% ANI or AAI value)	Genome size (bp)	No. of reads (millions)	No. of contigs	*N*_50_ (kbp)	G+C content (mol %)	No. of CDS^*a*^	Coverage (×)
*Bacillus infantis* 10Pi	SEHK00000000	SRR8492865	Bacillus infantis NRRL B-14911 (98.46% ANI)	5,001,688	1.42	32	339	45.6	5,619	137
Flavobacteriaceae 144Ye	SEHM00000000	SRR8492866	Seonamhaeicola sp. strain S2-3 (65.48% AAI)	3,304,492	1.80	32	406	33.3	3,028	261
Dermacoccaceae 147Ba	SEHL00000000	SRR8492864	Dermacoccus nishinomiyaensis M25 (75.77% AAI)	3,157,631	1.17	26	279	68.2	3,019	180
Idiomarina sp. strain 29L	SDJS00000000	SRR8491236	Idiomarina piscisalsi 10PY1A (94.52% ANI)	2,558,893	1.44	7	767	47.4	2,415	273
Vibrio alginolyticus Br233	SMLJ00000000	SRR8648834	*Vibrio alginolyticus* ATCC 33787 (98.57% ANI)	5,053,409	1.28	50	418	44.7	4,630	125
Vibrio owensii Br345	SMLI00000000	SRR8648835	*Vibrio owensii* XSBZ03 (96.84% ANI)	5,812,423	1.12	30	605	45.7	5,266	94

a CDS, coding sequences.

MiGA analysis revealed, based on the ANI or AAI values, that three of the isolates are candidate novel species, while three other species are candidate novel strains (Table 1). A total of 26 gene clusters for putative biosynthetic secondary metabolites from all of the genomes combined were predicted using antiSMASH. The understanding, manipulation, and elucidation of these genomic elements can help in the discovery of novel marine bioactive natural products.

### Data availability.

The genome sequences described here have been deposited in DDBJ/ENA/GenBank under the accession numbers and Sequence Read Archive (SRA) numbers listed in Table 1.
